# Perspectives and limits of cancer treatment in an oldest old population

**DOI:** 10.1007/s40520-021-01821-2

**Published:** 2021-03-11

**Authors:** Beatrice Di Capua, Andrea Bellieni, Domenico Fusco, Maria Antonietta Gambacorta, Luca Tagliaferri, Emanuele Rocco Villani, Roberto Bernabei, Vincenzo Valentini, Giuseppe Ferdinando Colloca

**Affiliations:** 1 UOC Radioterapia Oncologica, Dipartimento di Diagnostica per Immagini, Radioterapia Oncologica ed Ematologia, Fondazione Policlinico Universitario Agostino Gemelli IRCCS, Rome, Italy; 2 Dipartimento di scienze dell’invecchiamento, neurologiche, ortopediche e della testa collo, Fondazione Policlinico Universitario Agostino Gemelli IRCCS, Università Cattolica del Sacro Cuore, sede di Roma, Largo A. Gemelli 8, 00168 Rome, Italy

**Keywords:** Oldest old, Geriatric oncology, Personalized medicine, Cancer, Elderly, Radiation oncology

## Abstract

**Background:**

Population of oldest old will grow dramatically in the next future and cancer, physiologically related to aging, will be very prevalent among them. Lack of evidence is a huge problem to manage cancer in oldest old and will be more and more in the next years.

**Aims:**

Our purpose was to investigate the characteristics of a population of oldest old patients with cancer treated in the Radiation Oncology Unit of Fondazione Policlinico A. Gemelli IRCCS.

**Methods:**

We conducted a retrospective study. The primary outcome was to evaluate which characteristics of the population could influence the choice of oncological treatment (with radical or non-radical intent).

**Results:**

We identified a total of 348 patients: 140 were on follow-up; 177 were under treatment; 31 were considered not eligible for treatments. Patients under treatment had a high comorbidity index (mean Charlson Comorbidity Index 5.4), and a high prevalence of polypharmacy (mean number of drugs 5.6). More than half (53.1%) was treated with radical intent. Patients treated with radical intent were 1 year younger (87.1 years old vs 88.1 years old), more performant (ECOG 0.7 vs 1.3), and had less prevalence of metastatic neoplasia (6.4% vs 34.9%); comorbidities and drugs did not show differences in the two groups.

**Conclusion:**

Oldest old, usually not considered in international guidelines, are treated for oncological disease, often with radical intent. The treatment seems not to be tailored considering comorbidities but on performance status.

## Introduction

The scientific community is preparing to the so-called “Silver Tsunami”: in the next future, the population of elderly people will grow, and it will deeply change the world and the healthcare scenario. People born in the years of the economic boom, “baby boomers”, are estimated to be 73 million to date in United States [1]^.^ These people are destined to enter and fill up in few years the group of oldest old. The term “oldest old” is used to refer to people aged 85 years or older. In United States this is the population group that is growing faster: it is expected by 2050 oldest old in United States they will triple in number.

Aging is characterized by high number of comorbidities and high prevalence of polypharmacy and disability. Moreover, cancer is deeply related to aging: by 2030 70% of all cancer diagnosis will be made in elderly. Aging is associated with fundamental changes in health status that makes elderly at high risk of being frail such as sense organs deficits, chronic disabling diseases [2], cognitive impairment and cancer, all conditions related to aging and frailty. Polypharmacotherapy is also very prevalent in older adults [3]^,^ it relates to drugs interactions and drugs adverse events, especially in oldest old with cancer [4].

Despite the rising interest in oldest old, they are rarely enrolled in clinical trial, because of their complexity, and this determine serious lack of evidence and specific guidelines, especially those regarding cancer [5–7]. It’s very difficult, to date, to make evidence-based decision on treatment for oldest old with cancer and predict response to treatments [8]. In the report “*Delivering High-Quality Cancer Care: Charting a New Course for a System in Crisis*” written in 2013 by the American Institute of Medicine, it is underlined the urgency of having evidence on elderly patient with cancer [8, 9]. Recently an important epidemiologic study made in United States on oncological patients aged 85 or older was published; the study showed that, to date, 8% of all new cancer diagnosis are made in the oldest old [10].

Oldest old are a great mystery for science: on one side they have more comorbidities and disabilities, on the other they seem to have some protective factors that reduce the risk of some diseases. In the next future, when baby boomers will join the group, we can imagine that this population will grow and change more and more. We must work now to be ready to these needs, especially in oncological field, since cancer will be a relevant issue for them. The aim of our study was to investigate the characteristics of a population of oldest old treated by the Radiation Oncology Unit in a University Hospital, and how these features affect the intent of the prescribed oncological treatment.

## Materials and methods

We conducted a retrospective study to investigate the characteristics of a population of oldest old patients treated in the Radiation Oncology Unit (Gemelli ART—Advanced Radiation Oncology) of Fondazione Policlinico A. Gemelli IRCCS involving also the patient treated with Interventional Radiotherapy (brachytherapy) at Interventional Oncology Center of the same institution [11]. We analyzed medical records of patients aged 85 years or older, which have been evaluated by the Radiation Oncology Unit from June 2018 to May 2019.

We separated the outpatients on follow-up (group A) from the patients treated with radio-oncological treatment (radiotherapy and/or chemotherapy and/or hormone therapy and/or surgery) (group B) and the patients considered not eligible for treatments at the time of the evaluation (group C).

For all patients, we collected socio-demographic data and data regarding cancer. For patients belonging to group B, we also collected data regarding type of treatment, toxicities developed, comorbidities, and drugs. A subgroup of patients from group B, did not undergo the treatment prescribed for personal choice or performed it in another hospital; for them, we could not collect data regarding toxicities.

Primary outcome was to evaluate which characteristic of the population could influence the choice of the oncological treatment (treatment with radical vs not radical intent).

For radical intent, we mean a treatment whose target is to eradicate cancer. Treatment with not radical intent may be palliative if its target is to control a specific symptom (pain, bleeding etc.) or local tumor control when the purpose is to slow down or interrupt as long as possible cancer growth.

### Statistical analysis

All data are presented as mean ± standard deviation (SD) or median and interquartile range (IQR) when referring to continuous variables and as frequency and related percentage when referring to categorical variables, unless otherwise specified. To detect differences between patients treated with radical intent and patients treated with not radical intent, continuous data were analyzed by Student’s *t* test or Mann–Whitney *U* test where appropriate. Categorical data were analyzed through Chi-square or Fisher exact test where appropriate. A *p* value < 0.05 was considered significant and reported as such. SPSS 20.0 (IBM Inc.) for Windows was used for all the analyses.

## Results

**Fig. 1:**
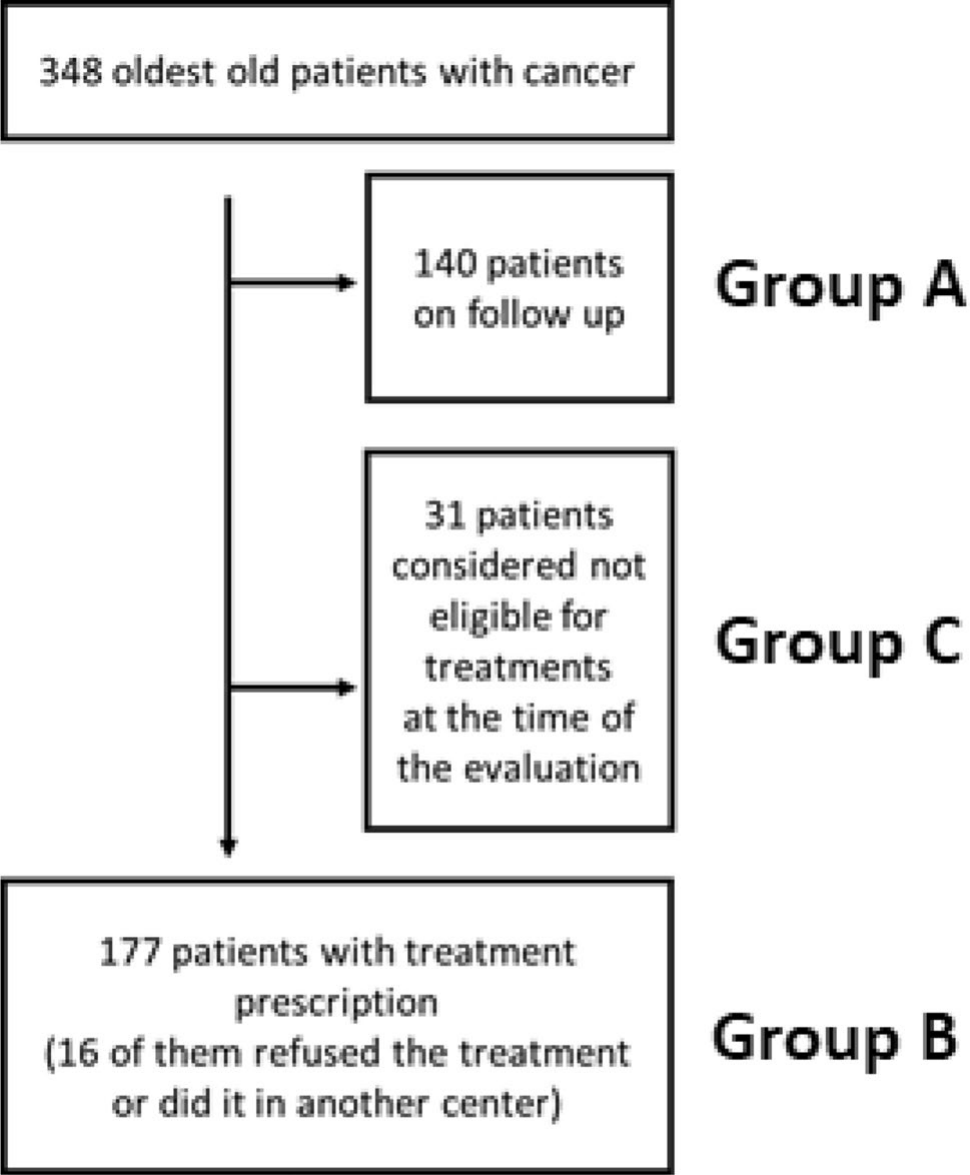
Flow chart of oldest old patients belonging to the Unit of Radiation Oncology

We identified a total of 348 patients that have been evaluated by the Unit of Radiation Oncology from June 2018 to May 2019. Within this group 140 were outpatients on follow-up (group A); 177 patients received a radio-oncological treatment (radiotherapy and/or chemotherapy and/or hormone therapy and/or surgery) (group B); 31 patients were considered not eligible for treatments at the time of the evaluation (group C) (Fig. 1).

Patients of group B received all a radio-oncological treatment. Someone was treated with radiotherapy alone or radiotherapy with concomitant chemotherapy or hormone therapy. For radiotherapy treatment have been included both patients went under external beam radiotherapy or interventional radiotherapy (brachytherapy). Moreover, patients who did a radiotherapy treatment for prostate or breast cancer continued to be followed up during hormone therapy if prescribed. We also decided to collect data on patients undergoing surgical procedures in the same year.

Among patients in group B, 161 completed the treatment in our Hospital, while 16 patients were treated in a different Hospital or decided not to undergo the prescribed therapy. Those oldest old patients were 5.3% of all patients treated at our center between June 2018 and May 2019. We focused our analysis on group B.

**Fig. 2:**
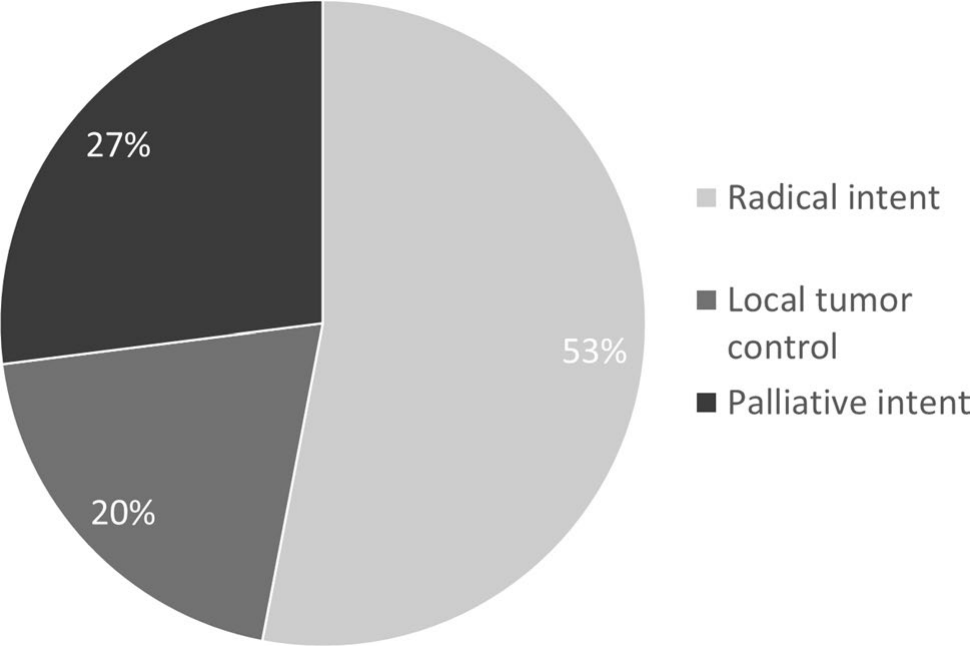
Distribution in the population of different treatments intents

Group B was homogenous for sex (50.8% male), and the mean age was 87.6 years old (minimum age 85 years old, maximum age 99 years old). The population had a high comorbidity index (mean Charlson Comorbidity Index 5.4, without considering cancer), and a high prevalence of polypharmacy (mean number of drugs 5.6). The mean value of ECOG (Eastern Cooperative Oncology Group) performance status was 1. 19.8% of patients were treated for a metastatic cancer (Table 1). Among the 177 patients, 129 (72.9%) were treated with radiotherapy alone, 60 (33.9%) were treated also with hormone therapy and 22 (12.4%) underwent to chemotherapy too. 18 patients (10.2%) had a surgical procedure in the previous year, of whom 10 underwent breast surgery, 4 underwent pulmonary lobectomy, 3 underwent gastrointestinal surgery. Regarding the purpose of the oncological treatment, 94 patients (53.1%) were treated with radical intent, 35 patients (19.8%) for local tumor control, and 48 patients (27.1%) with palliative intent (Fig. 2). It is important to underline that patients undergoing palliative treatment were not necessarily terminal.

**Table 1:** General characteristics of patients undergoing treatment

Population	177 patients (100%)
Male sex (*n*, %)	90 (50.8%)
Age (years) (mean, SD)	87.6
CCI (mean, SD)	5.4
ECOG (mean, SD)	1.0
Number of drugs (mean, SD)	5.6
Metastatic disease (*n*, %)	35 (19.8%)
Radiotherapy (*n*, %)	129 (72.9%)
Hormone therapy (*n*, %)	60 (33.9%)
Chemotherapy (*n*, %)	22 (12.4%)
Surgery (*n*, %)	18 (10.2%)
Radical intent (*n*, %)	94 (53.1%)
Local tumor control intent (*n*, %)	35 (19.8%)
Palliative intent (*n*, %)	48 (27.1%)
Geriatric Consultation (*n*, %)	27 (15.3%)

*CCI* Charlson Comorbidity Index

Breast cancer was the most prevalent cancer (18.6%), followed by prostate cancer (16.4%), skin cancer (13.6%), lung cancer (9.0%), colon rectal cancer (9.0%), kidney and urinary tract cancer (7.9%), gynecological cancer (6.8%) and head and neck cancer (6.2%) (Table 2).

**Table 2:** Prevalence of tumor site in patients undergoing treatments

Breast	33 (18.6%)
Prostate	29 (16.4%)
Skin	24 (13.6%)
Lung	16 (9.0%)
Colon and rectum	16 (9.0%)
Urologic	14 (7.9%)
Gynecologic	12 (6.8%)
Head and neck	11 (6.2%)
Liver and biliary tract	8 (4.5%)
Soft tissue	7 (4.0%)
Lymphoma	3 (1.7%)
Brain	2 (1.1%)
Eye	1 (0.6%)
Esophagus	1 (0.6%)

In this group, only 27 patients (15.3%) had a formal onco-geriatric consultation; 74.1% of geriatric consultations were asked for comprehensive geriatric assessment and clinical evaluation before starting the treatment, 25.1% were asked for a clinical problem or an emerging symptom (Table 1).

An overall acute and/or subacute toxicity was detected in only 10.6% of patients (17 on 161 patient); in two cases the toxicity was relevant (grade 3, cutaneous toxicity). In the other cases, there were hematological, cutaneous, neurological or gastrointestinal toxicity of grade 1 or 2. Six patients interrupted the treatment for toxicity or for other clinical reasons; one of them restarted the treatment. One patient died during the treatment.

We divided patients who were undergoing oncological treatment in two groups and compared them: the first group (94 patients) includes patients who were treated with radical intent, the second group (83 patients) includes patients treated with not radical intent (local tumor control or palliative intent). These two groups were compared for general characteristics, type of treatment, treatment intent and tumor site (Table 3). The two groups were homogeneous for sex and number of drugs taken. Patients treated with radical intent were one year younger (mean age 87.13 years old vs 88.08 years old; *p* = 0.024; difference between the two groups means − 0.96; 95% CI − 1.76 to − 0.15, data not shown), showed better functional status (mean ECOG 0.7 vs 1.3; *p* < 0.001; difference between the two groups means − 0.6; 95% CI − 0.88 to − 0.31, data not shown) and had less prevalence of metastatic neoplasia (6.4% vs 34.9%; *p* < 0.001; absolute reduction of risk − 81.2%, 95% CI − 69.1 to − 89.3%) than those treated without radical intent. Radiotherapy was less frequently prescribed to patients treated with radical intent, being the estimated proportion for radical intent 53.2% (95% CI from 45.3% to 64.9%) while the estimated proportion for palliative intent where 92% (95% CI from 85.1% to 96.6%), with a *p* value < 0.001. Those data lead to an absolute reduction of radiotherapy use of − 37.4% (95% CI − 48.2 to − 25.1%) between patient treated with radical intent vs those treated with palliative intent. On the other hand, hormone therapy and surgery were more frequently prescribed for those treated with radical intent. Breast and prostate cancers were more prevalent in the group of patients treated with radical intent (breast cancer 29.8% vs 6.0%, *p* < 0.001; prostate cancer 23.4% vs 8.4%; *p* = 0.007), while lung cancer was more prevalent in those treated with not radical intent (4.3% vs 14.45%; *p* = 0.033).

**Table 3:** Comparison between patients treated with radical and not radical intent

	Radical intent (*n* = 94)	Palliative intent/local tumor control (*n* = 83)	*p* value
Male sex (*n*, %)	45.7% (43)	56.6% (47)	0.148
Age (median, IQR)	87 (86–88)	87 (86–90)	*0.181*
Age (mean, SD)	87.13 (2.05)	88.08 (3.29)	0.024
Charlson comorbidity Index (mean, SD)	5.22 (1.1655)	5.67 (1.5333)	0.055
ECOG (mean, SD)	0.7 (0.5517)	1.3 (0.8786)	< *0.001*
Number of drugs (DS)	5.3 (2.9808)	5.9 (3.0374)	0.268
Metastatic disease (n M1)	6.4% (6)	34.9% (29)	< *0.001*
Radiotherapy (*n*, %)	55.3% (52)	92.8% (77)	< *0.001*
Chemotherapy (*n*, %)	16.0% (15)	8.4% (7)	0.130
Hormone therapy (*n*, %)	51.1% (48)	14.5% (12)	< *0.001*
Surgery (*n*, %)	18.1% (17)	1.2% (1)	< *0.001*
Interrupted treatment (*n*, %)	5.0% (4)	1.5% (1)	0.32
CGA (*n*, %)	21.1% (19)	9.6% (8)	0.06
Breast (*n*, %)	29.8% (28)	6.0% (5)	< *0.001*
Prostate (*n*, %)	23.4% (22)	8.4% (7)	*0.007*
Lung (*n*, %)	4.3% (4)	14.5% (12)	*0.033*
Skin (*n*, %)	11.7% (11)	15.7% (13)	0.442
Urologic (*n*, %)	2.1% (2)	13.3% (11)	0.07
Head and neck (*n*, %)	2.1% (2)	10.8% (9)	0.12
Gynecologic (*n*, %)	9.6% (9)	3.6% (3)	0.21
Esophagus and stomach (*n*, %)	0% (0)	1.2% (1)	0.87
Colon and rectum (*n*, %)	6.4% (6)	12.0% (10)	0.26
Liver and biliary tract (*n*, %)	2.1% (2)	7.2% (6)	0.32
Brain (*n*, %)	1.1% (1)	1.2% (1)	0.95

*p*-value for distribution of the variable between frail and non-frail patients is in italic when significant at < 0.05 level

## Discussion

The first important result of our study is that, despite the lack of specific indications in international guidelines, a high number of oldest old undergoes oncological treatments in our department. The second important result is that more than half of them received a treatment with radical intent.

In all our sample, the treatment more frequently proposed was, of course, radiotherapy, since our population belongs to this Unit. The high number of oldest old patients treated with radiotherapy confirms the evidence that this is an ideal therapy for older adults [12, 13]. The second more frequent treatment was hormone therapy, because of the large prevalence of breast and prostate cancer. In oldest old women, the prescription of hormone therapy as adjuvant therapy in patients who undergo surgery, should be well evaluated, according to functional status and life expectancy [14–16]. Although hormone therapy is considered a safe and well-tolerated therapy, the possible side effects must be evaluated and followed to avoid complications: hormonal blockade can have negative effects on cognitive, metabolic, and muscular systems [17–23]. Considering the importance of these syndromes on elderly people, it will become more and more urgent in the future to have strong evidence available to improve support therapy. The modern society will soon face to a new scenario, a world with a high number of oldest old patients that will probably be generally healthier than previous generations, but with a high prevalence of cancer disease.

We also observed, despite the age and the lack of specific indications on guidelines, that a considerable part of our population underwent chemotherapy and surgery. For what concerns chemotherapy, the population is rather heterogeneous, with a prevalence of colon rectal cancer and gynecological neoplasia; for surgery, the most prevalent tumors were breast cancer and gynecological cancer. We know that breast surgery, to date, is characterized by noninvasive techniques that can assure a radical target in early stages and can be safely offered to frailty patients.

Our population of treated patients was homogeneous for sex. We know indeed that women are more long-lived but they get old with more disabilities [24], so it is possible that they are considered less frequently for an oncological treatment. The mean age of our population was high, 87 years old, and the maximum age was 99 years old.

The characteristics of the population in treatment are accordant with literature: the population is characterized by a high comorbidity index (mean CCI 5.4 without considering the tumor) and high prevalence of polypharmacotherapy (almost half of the patients took five drugs or more) [2]. These two characteristics are homogeneous between the group of patients treated with radical intent and patients treated without radical intent. These data suggest that neither the number of comorbidities, neither the number of drugs is considered in the choice, whereas performance status and disease stage are the fundamental elements taken into consideration by radiation oncologists to design the treatment plan. ECOG and disease stage are indeed the two parameters with which the oncologists have more confidence. Nevertheless, we should consider that comorbidities and polypharmacy increase the risk of side effect during oncological treatments and drugs interactions: gastro-enteric toxicity and dehydration in patients with cardiological and kidney disease, the use of corticosteroids in patients affected by type 2 diabetes mellitus, hypertension, osteoporosis or gastropathies, or the use in this population of antiplatelet agents, anticoagulants, hypoglycemic agents, diuretics, as well as vitamin D [25]. Liver and kidney failure can modify considerably chemotherapy metabolism with higher risk of toxicity and drugs interactions [26, 27]

It is important to underline that the high prevalence of polypharmacotherapy is relevant for geriatric patients, because related to drugs interactions, inappropriate prescription, adverse events, and mortality. The prevalence of polypharmacy in our population is similar to general population of oldest old [28, 29].

We also investigated the prevalence of treatment-related toxicities that was quite low. Toxicities detected were low-grade toxicities but could be considered important for a frail patient. The low prevalence of toxicity can be explain in part by the fact that can be under reported in clinical records, but more likely by the fact that our center use the most recent technologies for radiotherapy, as Volumetric Modulated Arc Therapy or Stereotactic Body Radiation Therapy [30]. A very reliable and positive data are, indeed, that only five patients interrupted the treatment, which means that 97% of patients completed the treatment. In several studies investigating the feasibility of chemotherapy in oldest old only 50% or less of patients could complete the chemotherapy planned [31, 32]. This difference suggests that radiotherapy is a treatment that fits perfectly to elderly with better predictable effects and higher possibility to complete the planned treatment.

As we mentioned earlier, comparing the group of patients treated with radical intent, and those treated without radical intent, we observed that the choice is made primarily on age, ECOG performance status, and metastatic disease. These data suggest that in oncology, also for the oldest old, functional status is considered one of the most important prognostic factors to take into account in therapeutic choices. Unfortunately, the only data we could collect on functional status was ECOG performance status that occurred in 102 records (57.6% of records). The mean ECOG of treated patients was 1, that means a slight impairment in functional status; the difference between the two groups was almost of one point. Although performance status is the most frequent tool in oncology for measuring functional status, it is important to underline that it cannot be considered an effective surrogate of comprehensive geriatric assessment [33]. Anyway, most of the screening tools for elder patient with cancer also include ECOG [34].

Radiotherapy is more prevalent in the group of patients treated with palliative intent. Radiotherapy indeed is a very flexible technique that allows to adapt intensity and duration of the treatment according to the purpose of the treatment and it is well tolerated in old and frail patients. [35–37]

Hormone therapy is more prevalent in the group of patients treated with radical intent, because is used as adjuvant therapy in breast and prostate cancer to avoid relapses.

In our study, 27 on 177 patients had a geriatric evaluation (15.3%). About ¾ of these consultations were asked for comprehensive geriatric assessment and evaluation of the treatment proposed by the oncologist. About ¼ of the consultations were asked for the management of a clinical acute condition occurred during or after the treatment. An interesting data is that the consultation is asked more frequently when the patient is candidate to have a treatment with radical intent, although it was not statistically significant, probably due to the small number of patients involved. Although the number of geriatric consultations found in patients’ records is quite low, in our department, the presence of a geriatrician specialized in geriatric oncology and mainly involved in the Radiotherapy ward, allowed to create a special care pathway for frail patients that can undergo some treatments in a protected and supported condition.

We also observed 140 oldest old patients that were in follow-up. This high number of patients is the evidence that cancer is no more a terminal disease, but it can be treated with success also in elderly.

The main strength of our study is that we could evaluate in a high number of oldest old the main characteristics of their oncological treatments in a department of radiotherapy.

The main limitation of our study is that, since we collected data retrospectively, we could not record more data on functional status then ECOG performance status; moreover, some data on cancer stage and toxicity lack. Lastly it was not possible to estimate how many patients were not directed to an evaluation of treatment by the care provider, and so what is the real proportion of oldest old treated for cancer.

## Conclusions

Our study shows how oldest old, usually not considered in international guidelines, are treated for oncological disease, often with radical intent. This result may be influenced by the fact that the university hospital from which the data were extrapolated represents a center of excellence with the constant presence of a geriatrician that is dedicated to oncological patients. The treatment seems not to be tailored considering comorbidities but on functional status, that is most of the time measured through performance status. Almost all patients completed the treatment without presenting serious toxicities.

We wish to have specific clinical trials tailored on oldest old, which will include comprehensive geriatric assessment and the evaluation of a multidisciplinary team for having robust data on tolerance and efficacy of new treatments on oldest old population.
